# Clinical Management of Chronic Pelvic Pain in Endometriosis Unresponsive to Conventional Therapy

**DOI:** 10.3390/jpm12010101

**Published:** 2022-01-13

**Authors:** Augusto Pereira, Manuel Herrero-Trujillano, Gema Vaquero, Lucia Fuentes, Sofia Gonzalez, Agustin Mendiola, Tirso Perez-Medina

**Affiliations:** 1 Department of Gynecologic Surgery, Puerta de Hierro University Hospital, 28222 Madrid, Spain; gemitava@gmail.com (G.V.); lucia.fuentes72@gmail.com (L.F.); tirsoperezmedina@gmail.com (T.P.-M.); 2 Department of Anesthesia, Puerta de Hierro University Hospital, 28222 Madrid, Spain; manuelherrerotrujillano@gmail.com (M.H.-T.); agustin.mendiola@salud.madrid.org (A.M.); 3 Department of Rehabilitation, Puerta de Hierro University Hospital, 28222 Madrid, Spain; sglopez@salud.madrid.org

**Keywords:** amitriptyline, botulinum toxin, diazepam, endometriosis, hypogastric plexus blockade, medical treatment, non-responders, pain, physiotherapy, pulsed radiofrequency

## Abstract

Background: Although several treatments are currently available for chronic pelvic pain, 30–60% of patients do not respond to them. Therefore, these therapeutic options require a better understanding of the mechanisms underlying endometriosis-induced pain. This study focuses on pain management after failure of conventional therapy. Methods: We reviewed clinical data from 46 patients with endometriosis and chronic pelvic pain unresponsive to conventional therapies at Puerta de Hierro University Hospital Madrid, Spain from 2018 to 2021. Demographic data, clinical and exploratory findings, treatment received, and outcomes were collected. Results: Median age was 41.5 years, and median pain intensity was VAS: 7.8/10. Nociceptive pain and neuropathic pain were identified in 98% and 70% of patients, respectively. The most common symptom was abdominal pain (78.2%) followed by pain with sexual intercourse (65.2%), rectal pain (52.1%), and urologic pain (36.9%). A total of 43% of patients responded to treatment with neuromodulators. Combined therapies for myofascial pain syndrome, as well as treatment of visceral pain with inferior or superior hypogastric plexus blocks, proved to be very beneficial. S3 pulsed radiofrequency (PRF) plus inferior hypogastric plexus block or botulinum toxin enabled us to prolong response time by more than 3.5 months. Conclusion: Treatment of the unresponsive patient should be interdisciplinary. Depending on the history and exploratory findings, therapy should preferably be combined with neuromodulators, myofascial pain therapies, and S3 PRF plus inferior hypogastric plexus blockade.

## 1. Introduction

Endometriosis is defined as an endometrial-like tissue that occurs outside the uterine cavity. Endometriosis is an estrogen-dependent, chronic inflammatory disease, and is the major contributor to chronic pelvic pain (CPP). Due to the difficulty of making an accurate differential diagnosis of this disease through symptoms [1], it might be difficult to establish a precise assessment of the disease’s prevalence and incidence. It is estimated that prevalence could be as high as 10% [2], with an annual incidence of 0.1% among women aged 15–49 years [3]. Overall, more than 80% of women diagnosed with CPP have endometriosis [4].

There are conventional therapeutic options for management of endometriosis, including medical treatment (non-hormonal and/or hormonal) [5,6] and surgery (conservative or definitive). Since 2019, a novel algorithm has been proposed for managing CPP in patients with endometriosis, including combined oral contraceptives (COCs), progestins or dienogest as first-line therapy, and gonadotropin-releasing hormone analogues (GnRHa) as second-line therapy [7]. Since all the available hormonal treatments interfere with ovulation, the desire for an active or future pregnancy must be considered when prescribing them [8]. In addition, these treatments are usually reserved for women in which surgery does not provide any benefit. It is estimated that the percentage of patients who do not respond to conventional treatment ranges from 30% to 60% [9,10]. Therefore, therapeutic options for the treatment of complex endometriosis-related pain require a better understanding of the mechanisms underlying endometriosis-induced pain. The likelihood of failure increases exponentially when the pain is due to central sensitization [11].

The aim of this study was to report our experience in the interdisciplinary management of women who suffer from endometriosis and CPP unresponsive to conventional therapies and who were seen in our hospital over the course of the past 3 years.

## 2. Materials and Methods

### 2.1. Patients

We reviewed clinical data from consecutive patients with endometriosis and CPP not responsive to conventional therapies at Puerta de Hierro University Hospital, Madrid, Spain from January 2018 to September 2021. Approval was granted by the Institutional Review Board. The main inclusion criterion was CPP and endometriosis patients who do not respond to conventional therapy persisting for at least 6 months. Conventional therapy for CPP was defined as medical treatment (non-hormonal and/or hormonal) [3,4] with first-line therapy being drugs such as combined oral contraceptives (COCs), progestins or dienogest; and second-line therapy being gonadotropin-releasing hormone analogues (GnRHa) and/or surgery (conservative or definitive). All patients were identified in endometriosis or CPP unit outpatient clinics. All non-responder patients were classified into neuropathic or nociceptive pain based on history, examination, and physician’s judgment. Regarding nociceptive pain, patients were distinguished if visceral (in the abdominal-pelvic viscera and visceral peritoneum) or somatic (in abdominal and pelvic wall, pelvic floor muscles, perineum, and parietal peritoneum).

### 2.2. Data Assessed

Data included a full history (gynecologic, obstetric, and surgical), comorbidities (painful bladder syndrome, migraine, fibromyalgia, and irritable bowel syndrome), pain in neighboring organs (bladder, rectum, dysmenorrhea, and dyspareunia), duration of pain (estimated by the patient as the number of years between onset of symptoms and date of first consultation), characteristics, localization and irradiation of pain, and finally, the intensity of pain, measured using a visual analogue scale (VAS) ranging between 0 (no pain) to 10 (worst pain ever experienced) [12].

### 2.3. Exploratory Procedures

Data were collected from exploratory findings, as our group reported in a previous study [13]:

(1) Neurologic examination S2–S4: (a) cotton swab test at the S2–S4 dermatome and vestibule; (b) assessment of the nerve’s motor response using the clitoris, bulbospongiosus, and perineal reflexes; (c) Tinel sign at the level of the sciatic spine to evaluate the third segment of the pudendal nerve [14]; (d) Tinel sign at the clitoris to evaluate the dorsal clitoris nerve [14].
(2) Exploration of the pelvic girdle: bilateral palpation to identify painful spots: retropubic, ischiopubic ramus, ischium, sacrospinous ligament, sacrum, and coccyx.
(3) Exploration of pelvic floor muscles: (a) Levator ani muscle (LAM): assessment of painful palpation of the pubococcygeus muscle; (b) Obturator internus muscle (OIM): Contracture of the IOM with flexion and external rotation of the hip in the supine decubitus position and transgluteal exploration of IOM segments: pelvic (ischium), medium (midpoint between the trochanter and coccyx) and gluteal (hip); (c) Piriformis muscle (PM): 5 cm above the medium segment of OIM.

During the examination, the patient was also asked to state the level of pain on a VAS scale of 0–10. The median pain intensity was calculated from the sum of all PN segments after exploration.

### 2.4. Protocols for Unresponsive Patients

Protocols for unresponsive patients used at our hospital were:

(a) Treatment with neuromodulators: amitriptyline as first-line agent and duloxetine, gabapentin, or pregabalin as second-line agent for 2 months, then dose adjustments, associations, or switch to another neuromodulator;
(b) Protocol 1 for the treatment of myofascial pelvic pain syndrome (somatic nociceptive), consisting of:
  - Physical therapy (including manual therapy), tissue mobilization, biofeedback, transcutaneous electrical nerve stimulation, or posterior tibial nerve stimulation;
  - Vaginal administration of 5 mg diazepam every 48 h, and maintenance of the treatment until initiation of physical therapy;
  - Trigger point injection (TPI) with 10 mL of 0.25% levo-bupivacaine, in case of improvement, for at least 3 months (30–50% pain relief). Repeated series of injections are considered when appropriate;
  - Levo-bupivacaine injection followed by 100 international units of onabotulinum toxin A (BTXA) (Botox^®^ by Allergan Inc., Irvine, CA). Each 100-unit vial is diluted in 10 mL of preservative-free saline, and 1 to 2 mL are injected EMG guided in bilateral LAM and OIM. Repeat injections are considered if the first series present without significant side effects, and if no sooner than 12 weeks;
(c) Protocol 2 for the treatment of visceral pain (nociceptive) with minor or major opioids combined with blockade of the inferior or superior hypogastric plexus (IHP or SHP);
(d) Protocol 3 for patients with neuromodulators and neuropathic pain with peripheral nerve blocks.
(e) Protocol 4: Pulsed radiofrequency (PRF) of S3 with IHP block for the treatment of neuropathic and nociceptive pain in the same way. The technique consists of using a 10 cm radiofrequency cannula with 10 mm active tip (G4™ RF Generator^®^ by Boston Scientific, Marlborough, MA) by means of a transsacral fluoroscopy-guided approach, entering the bilateral posterior S3 foramina, until a positive sensory (< 0.5 V) and motor stimulation (> 0.8 V) is attained, aided with a lateral X-ray to avoid entering the pelvic viscera. At this point, a PRF lesion is performed (45 V, 240 s) and then the tip of the cannula is slightly introduced towards the anterior surface of the sacrum. Finally, contrast dye is used to identify the spreading around the anterior surface, avoiding vessel and viscera images. Subsequently, the blockade (Betamethasone 12 mg and L-Bupivacaine 0.25% 10 cc) is administered, distributed through the two cannulas.

Patients were followed up in the pain unit, rehabilitation, and endometriosis consultations. A positive response if there had been a benefit in symptom relief higher than 30% was considered.

## 3. Results

A total of 46 patients with endometriosis were confirmed to have CPP unresponsive to conventional treatment. Demographic characteristics of the study population are shown in Table 1.

The median maternal age was 41.5 years (range, 25–52) and the median VAS pain intensity was 7.8/10 (range, 5.5–10). The most common symptoms were abdominal pain, located in the hypogastrium and/or iliac fossae. Pain during sexual intercourse was reported by 65.2% of patients, proctalgia by 52.1%, and urologic pain by 36.9%. A total of 73.9% of patients had associated comorbidities: 4.3% painful bladder syndrome, 6.5% migraine, 15.2% fibromyalgia, and 17.4% irritable bowel syndrome. Twenty-six out of 46 patients who did not respond to conventional therapy were examined in the chronic pelvic pain unit to obtain a complete map of the location of the pain. The results were as follows:

The overall sensory deficit at S2–S4 was 17 out of 26 endometriosis patients. A total of 23 patients from the entire cohort had a pudendal nerve motor deficit, and 18 of 26 patients presented a positive cotton swab test at the vestibule. Exploration of the third segment of the pudendal nerve revealed pain in 22 patients, and 3 out of 26 patients had pain localized in the dorsal clitoris nerve. Of the 26 patients, 25, 24, and 18 presented pain localized to the LAM, OIM, and PM, respectively. There were 18 patients with pain in all pelvic muscles (MEA, MOI, and MP) and only one patient was without muscle pain. Clinical and exploratory findings are shown in Table 2.

After history and examination of the unresponsive patients with endometriosis, findings only compatible with nociceptive pain were identified in 26% of patients (somatic: 9; visceral: 3). Only 2% had neuropathic pain, and 62% had mixed symptoms (nociceptive and neuropathic). The treatment administered to non-responding patients was according to type of pain. Results are shown in Table 3.

Five out of 6 patients responded to botulinum toxin, obtaining a median pain symptoms relief of 40% (range, 30–90%) and median follow-up was 3.5 months (range, 2.2–6.3 months). Seven of the 33 patients with nociceptive and neuropathic pain were treated with IHP block associated with PRF of S3. The median pain relief obtained was 50% (range, 50–70%) and median follow-up was 3.8 months (range, 1.5–7.7 months). The use of neuromodulators including tricyclic antidepressants (TCAs) provided a benefit of 43% (12 responders out of 28) and 50% (11 out of 22), respectively.

## 4. Discussion

Conventional treatment for pain management in women with endometriosis can be summarized in three options: analgesics, which include non-steroidal analgesics (NSAIDs), hormonal agents, and surgical treatment. Treatment decisions are individualized and tailored to pain characteristics (intensity, clinical presentation, extent and location), reproductive desires, age, medication side effects or contraindications, final results of surgery, and cost of treatment).

Apparently, good results in relieving endometriosis-related pain should be expected with conventional treatments. For example, success in pain reduction should be achieved through the administration of progestogens [14,15,16,17], just as the pro-inflammatory response should be controllable with NSAIDs. Therefore, if endometriotic lesions contribute to pain, their surgical removal or destruction should relieve pain [18].

Unfortunately, 25–33% of patients who undergo hormonal treatments do not achieve pain relief [19,20,21,22], either due to intolerance or contraindications to the therapy. In addition, the response to NSAIDs is often ineffective [5]. In the case of surgery, it does not reduce pain in 20–28%, [23,24], failing in 50% of patients before 1 year [18] and requiring another surgery within 2 to 5 years in 25.5% and 40–50% of the cases, respectively [10]. There are also rare events such as progesterone resistance, relief of pain symptoms after GnRHa therapy in patients who did not previously respond to progesterone treatment [22,25], or pain recurrences after surgery in the absence of new lesions [26].

If we delve deeper into the pathophysiology of CPP in endometriosis, we find a complex field that justifies the lack of response to different treatment options. The pathophysiology of CPP in endometriosis is an evolutive process initiated by an inflammatory response in the peritoneal fluid, which facilitates growth of endometrial-like tissue and generates a process of neuro-angiogenesis in endometriotic lesions. A direct activation of sensory nerve endings results in neurogenic inflammation, which leads to stimulation and activation of peripheral nerve endings, causing peripheral sensitization followed by central sensitization. In addition, a sensitization of multiple organ afferents can induce cross-organ sensitization.

Other pain-related syndromes associated with peripheral and central sensitization, such as irritable bowel syndrome, painful bladder syndrome, fibromyalgia, and migraine, coexist in patients with endometriosis [27,28,29,30,31]. It is also common to find psychic disorders such as depression, anxiety, and chronic fatigue [32,33]. In these patients with comorbidities, one pain may predominate over another, amplifying the painful signal to another organ, which is an indication of the presence of a neurologic overlap. In addition, in most women with endometriosis and CPP, there is some concomitant myofascial dysfunction [34].

Most medications used for non-responder endometriosis patients are not a US FDA-approved indication, although most of the evidence supports the safety and efficacy of those treatments in women with pelvic pain syndromes. Our results have revealed partial improvements in 70% of non-responder patients with any of the aforementioned therapies.

In the face of such a bleak outlook, the medical management of endometriosis-related CPP should be integrated into an interdisciplinary approach. Before starting treatment in patients unresponsive to conventional therapy, all women should undergo a thorough examination, being classified as:

### 4.1. Patient with Neuropathic Pain

For CPP unresponsive to targeted therapy, neuromodulators are widely accepted in the field of pain medicine, since, in this case, altered central pain processing adopts a significant role. Most of our patients (88%) were treated with neuromodulators, with a response rate of 43%. The TCAs are often considered the first-line treatment for centralized pain conditions and the treatment of neuropathic pain. There are no studies in which the use of amitriptyline has been researched in endometriosis. However, at least one multicenter, placebo-controlled trial has studied this compound in patients with female interstitial cystitis/painful bladder, reporting a moderate to marked improvement (55%) at 12 weeks of treatment, in the absence of significant side effects [35]. In our study, TCAs in an unresponsive patient relieve 50% of the symptoms. If the patient is unable to reduce pain through a therapeutic dose or has any intolerance, the next step should be taken (gabapentin, pregabalin or duloxetine).

After examination of the unresponsive patients, we identified that 85% and 12% of them complained of pain at the pudendal canal entrance and in the DCN, respectively. For women with significant pain symptoms, neuromodulators can be combined with peripheral nerve blocks (PNBs) and/or muscle relaxants. A PNB intervention that leads to a successful pain reduction supports the diagnosis of the disease, which happened in two-thirds of our patients. In order to offer long-term relief, PRF at PN or S3 should be recommended.

### 4.2. Patient with Nociceptive Pain

#### 4.2.1. Visceral Pain: Abdominal-Pelvic Viscera and Visceral Peritoneum

Once neuromodulators have been used, it is important to emphasize that the patient should continue to explore nonpharmacologic treatments that improve her symptoms whilst she is progressing through the subsequent lines of therapies. Tramadol should be used cautiously when selective serotonin uptake inhibitors and TCAs are taken concurrently. Long-term opioid therapy in CPP is not recommended, since it has potential side effects, it is highly addictive, and has no proven benefit [36,37,38,39,40] as we have reported in our study (Table 3). Short-acting opioids (oxycodone) should be limited for postoperative pain control or brief flare-ups of pain.

Visceral pain reflects afferent input traveling with autonomic nerves, i.e., with sympathetic or parasympathetic nerves. The pelvic area is innervated by a mixture of sympathetic, parasympathetic, and somatic networks. Sympathetic innervation emerges from T12 to L2 spinal cord segments, carried through the SHP that contains afferent pain fibers from the endometrium and other pelvic organs. Parasympathetic innervation travels through the sacral roots that meet the preganglionic fibers descending from the SHP and join the hypogastric nerves that converge into the IHP. In addition, somatic efferent and afferent innervation to the endometrium comes from the S2–S4 spinal cord.

More than half our patients were treated for visceral pain through the combination of minor opioids and/or sympathetic nerve blocks, which led to an overall response rate of 54%. Recent literature supports that SHP blockade can be an effective procedure for pain control and improvement of quality of life during refractory endometriosis [41]. More recently, Hetta DF et al. [42] combined SHP neurolysis with PRF of the sacral roots to improve the success rate of SHP neurolysis to control of pelvic and perineal cancer pain.

In addition to these publications, there are some studies regarding the efficacy of IHP blockade as a new target to relieve pain from the lower pelvic organs and genitalia [43]. Schultz [44] reported a novel technique using a fluoroscopy-guided transsacral approach towards the medial interior edge of the ventral sacral foramen to reach the IHP.

In our study, we observed similar results with SHP and IHP blocks (71% response) in comparison to previously published studies. In this regard, we have found a window of opportunity for patients unresponsive to conventional therapy with simultaneous visceral and neuropathic pain. For these patients, we used IHP blockade plus PRF of S3, because the access route is the same and because it decreases the pain rate by up to half over a period of approximately 4 months. Future research should assess the efficacy of this route.

#### 4.2.2. Somatic Pain: Abdominal and Pelvic Wall, Pelvic Floor Muscles, Perineum, and Parietal Peritoneum

Prolonged muscle contraction, spasm, and inappropriately high muscle tone are hypothesized to diminish blood supply and increase oxygen demand of the pelvic floor muscles. Ischemic muscle may secrete pain-producing substances, which further sensitize muscle nociceptors, alter receptor field properties, and convert wide-band mechanoreceptors into nociceptors.

Data supporting pelvic floor physiotherapy in CPP is limited. There are some studies on myofascial pelvic pain syndrome, painful bladder syndrome, and coccygodynia with improvement observed in patients [45,46,47,48,49,50,51,52,53,54]. Progressive muscle relaxation techniques for 12 weeks have shown a significant improvement in anxiety and depression levels in patients with endometriosis [55].

Several studies have reported that patients with pelvic floor myofascial pain might benefit from vaginal administration of 5 mg diazepam, while others see little or no benefit [56,57,58,59].

TPI is the procedure to treat focal, hypersensitive nodules within muscles that are markedly painful and may contribute to the development of central pain amplification. TPIs have revealed efficacy in a variety of neuromuscular pain syndromes and elevator ani pain [60,61,62,63,64].

However, BTXA in the pelvic floor is not a US FDA approved indication, but evidence supports the fact that it may reduce both general and myofascial pelvic pain and may be helpful for some women with CPP-related endometriosis [65,66,67,68,69,70]. A meta-analysis of CPP women treated with BTXA injections of the pelvic floor revealed a statistically significant decrease in pain scores [70].

Most patients tolerate injections in the office setting. Side effects of BTXA during the first 5 days may include flu-like symptoms, constipation, urinary retention or incontinence, and fecal incontinence [70]. All of these side effects were gradually resolved over approximately 3 months as the effects of BTXA wore off.

Ninety-six percent of patients with endometriosis who did not respond to conventional therapy had myofascial pelvic pain syndrome. The vast majority of them received a combined therapy for pelvic pain (physical therapy and/or vaginal diazepam and/or TPI and/or BTXA). Vaginal diazepam, TPI, or BTXA may facilitate physical therapy if the focal area of pain is small. Sixty percent of non-responder patients could benefit from the combined therapy. In addition, while TPI or BTXA presented a response rate in 70%–80% of patients, the use of BTXA can provide up to 40% symptom relief over a period of 3.5 months. Additional prospective studies are needed to confirm this finding.

Therefore, future research should be directed at improving early diagnosis and promoting any effective treatment for endometriosis. The delays in the diagnosis lead to the development of complex pain mechanisms and the lack of response to the treatment. Although an early search for effective treatments could prevent central pain sensitization, a multidisciplinary and multimodal approach focusing on patient history and examination is necessary.

## 5. Conclusions

In conclusion, all of the procedures reported above caused partial and transitory benefit to these patients, but they showed a clear benefit to quality of life in women with no response to conventional therapy. First-line treatment would comprise the administration of neuromodulators and, in case of a low response, a switch to or combination with another agent. Combined therapies have shown efficacy in the treatment of myofascial pain syndrome. Finally, there is the possibility of prolonging the pain-free period with botulinum toxin administration or IHP blocks associated with S3 RFP.

## Figures and Tables

**Table 1:** Demographic characteristics of patients with endometriosis patients unresponsive to conventional therapy.

Demographic Characteristics	
Age, Median Years (Range)	41.5 (25–52)
Pain intensity, median VAS ^1^ (range)	7.8 (5.5–10.0)
Duration of pain, years, median (range)	4 (1–26)
Comorbidities, patient number (%)	34 (74)
Painful bladder syndrome	2 (4.3)
Migraine	3 (6.5)
Fibromyalgia	7 (15.2)
Irritable bowel syndrome	8 (17.4)
Arthritis/arthralgia:	5 (11)
Parity, patient number (%)	
Nulliparous	22 (48)
Primiparous/Multiparous	24 (52)
Cesarean, patient number (%)	8 (17)
Surgery, patient number (%)	39 (84.8)
Pain location, patient number (%)	
Abdominal pain	36 (78.2)
Pain during sexual intercourse	30 (65.2)
Proctalgia	24 (52.1)
Urologic pain	17 (36.9)

^1^ VAS, Visual Analog Scale.

**Table 2:** Descriptive analyses with examination findings of endometriosis patients unresponsive to conventional therapy.

Exploratory Findings	*n* (%)
Sensory deficit at S2–S4	17 (65)
Q tip test at vestibule	18 (69)
Negative reflexes	23 (50)
Pain at peripheral nerves	23 (89)
Pain at third segment of the pudendal nerve	22 (85)
Pain at dorsal clitoris nerve	3 (12)
Pain at pelvic muscles	25 (96)
Levator ani	25 (96)
Obturator internus	24 (92)
Medium segment	12 (46)
Pelvic segment	12 (46)
Ischium segment	13 (50)
Piriformis	18 (69)
Pain at pelvic girdle	26 (100)
Retropubic	20 (77)
Ischiopubic ramus	17 (65)
Ischium	12 (46)
Sacrospinous ligament	18 (69)
Sacrum	7 (15)
Coccyx	17 (65)

**Table 3:** Descriptive analyses with examination findings of endometriosis patients unresponsive to conventional therapy.

Type of Pain	Treatment	*n* (%)	Responders, *n* (%)
All patients	Any kind of procedure	46 (100)	32 (70)
Nociceptive		45 (98)	31 (69)
Somatic		37 (80)	22 (60)
	Muscle relaxants	17 (46)	9 (53)
	Physiotherapy	16 (43)	8 (50)
	Trigger point injection	11 (30)	8 (73)
	Botulinum toxin	6 (16)	5 (83)
Visceral		22 (48)	13 (54)
	Minor opioids	7 (29)	4 (57)
	Major opioids	4 (17)	1 (25)
	Inferior hypogastric plexus block	-	-
	Superior hypogastric plexus block	4 (17)	2 (50)
Neuropathic		32 (70)	14 (44)
	Neuromodulators	28 (88)	12 (43)
	Neuromodulators: 1st line	22 (69)	9 (41)
	Neuromodulators: 2nd line	12 (38)	3 (25)
	Peripheral nerve block	5 (16)	2 (40)
	Pulsed radiofrequency: S3/Pudendal nerve	3 (9)	2 (67)
Mixed		33 (72)	22 (67)
Neuropathic + Nociceptive	Inferior hypogastric plexus blocks + S3 pulsed radiofrequency	7 (21)	5 (71)

## Data Availability

Data presented in this manuscript are available from the corresponding authors on reasonable request.
